# New York College of Veterinary Surgery

**Published:** 1860-01

**Authors:** 


NEW YORK COLLEGE OF VETERINARY SURGERY.—A college of this kind has recently been established in the City of New York, in an edifice specially erected for the purpose at the corner of Twenty-third Street and Sixth Avenue. It was chartered last winter by the Legislature, its projector being John Campbell Ralston, M.R.C.V.S., titles which we suppose mean Member of the Royal College of Veterinary Surgery of England. Mr. Ralston is Professor of the Theory and Practice of Veterinary Medicine; Mr. John Busteed, Professor of Anatomy and Surgery; and Dr. R. O. Doremus, Professor of Chemistry and Materia Medica.

We are rejoiced to find that the subject of Veterinary Medicine and Surgery is everywhere attracting the attention of the people of this country. There is no portion of the globe where there is so much need of well-regulated veterinary schools and hospitals as in the United States, for there is none where there is so much fine stock, and where so many noble animals daily die for the want of proper treatment.

